# Effects of physical exercise during adjuvant chemotherapy for breast cancer on long-term tested and perceived cognition: results of a pragmatic follow-up study

**DOI:** 10.1007/s10549-023-07220-7

**Published:** 2024-01-29

**Authors:** Willeke R. Naaktgeboren, Emmie W. Koevoets, Martijn M. Stuiver, Wim H. van Harten, Neil K. Aaronson, Elsken van der Wall, Miranda Velthuis, Gabe Sonke, Sanne B. Schagen, Wim G. Groen, Anne M. May

**Affiliations:** 1 Division of Psychosocial Research and Epidemiology, The Netherlands Cancer Institute, Amsterdam, The Netherlands; 2 Julius Center for Health Sciences and Primary Care, University Medical Center Utrecht, Utrecht University, Utrecht, The Netherlands; 3 Center for Quality of Life, The Netherlands Cancer Institute, Amsterdam, The Netherlands; 4 Centre of Expertise Urban Vitality, Faculty of Health, Amsterdam University of Applied Sciences, Amsterdam, The Netherlands; 5 Department of Health Technology and Services Research, University of Twente, Enschede, The Netherlands; 6 Rijnstate Hospital, Arnhem, The Netherlands; 7 Department of Medical Oncology, UMC Utrecht, Utrecht, The Netherlands; 8 Netherlands Comprehensive Cancer Organisation, Utrecht, The Netherlands; 9 Department of Medical Oncology, The Netherlands Cancer Institute, Amsterdam, The Netherlands; 10 Brain and Cognition Group, University of Amsterdam, Amsterdam, The Netherlands; 11 Department of Medicine for Older People, Amsterdam UMC, Vrije Universiteit Amsterdam, Amsterdam, The Netherlands; 12 Aging & Later Life, Amsterdam Public Health Research Institute, Amsterdam, The Netherlands; 13 Amsterdam Movement Sciences, Ageing & Vitality, Rehabilitation & Development, Amsterdam, The Netherlands

**Keywords:** Breast cancer, Cognition, Exercise, Physical activity

## Abstract

**Purpose:**

Cancer-related cognitive impairment (CRCI) following chemotherapy is commonly reported in breast cancer survivors, even years after treatment. Data from preclinical studies suggest that exercise during chemotherapy may prevent or diminish cognitive problems; however, clinical data are scarce.

**Methods:**

This is a pragmatic follow-up study of two original randomized trials, which compares breast cancer patients randomized to exercise during chemotherapy to non-exercise controls 8.5 years post-treatment. Cognitive outcomes include an online neuropsychological test battery and self-reported cognitive complaints. Cognitive performance was compared to normative data and expressed as age-adjusted z-scores.

**Results:**

A total of 143 patients participated in the online cognitive testing. Overall, cognitive performance was mildly impaired on some, but not all, cognitive domains, with no significant differences between groups. Clinically relevant cognitive impairment was present in 25% to 40% of all participants, regardless of study group. We observed no statistically significant effect of exercise, or being physically active during chemotherapy, on long-term cognitive performance or self-reported cognition, except for the task reaction time, which favored the control group (β = -2.04, 95% confidence interval: -38.48; -2.38). We observed no significant association between self-reported higher physical activity levels during chemotherapy or at follow-up and better cognitive outcomes.

**Conclusion:**

In this pragmatic follow-up study, exercising and being overall more physically active during or after adjuvant chemotherapy for breast cancer was not associated with better tested or self-reported cognitive functioning, on average, 8.5 years after treatment. Future prospective studies are needed to document the complex relationship between exercise and CRCI in cancer survivors.

**Supplementary Information:**

The online version contains supplementary material available at 10.1007/s10549-023-07220-7.

## Introduction

Over the last decades, the number of individuals living with and beyond a breast cancer diagnosis has increased [1–3]. Projections forecast that the population of cancer survivors will continue to grow in future years [4]. In this context, adequate care of cancer (therapy)-related side effects is increasingly important.

Cancer-related cognitive impairment (CRCI) is among the most common and burdensome side-effects in both breast cancer patients and survivors. The prevalence of CRCI varies widely across studies, with a mean prevalence of 44% for self-reported CRCI [5]. Prior research has reported that effects on cognitive performance can be detected even 20 years after treatment [6]. The pathophysiology of CRCI is multifactorial, with key roles for (anthracycline-based) chemotherapy, having cancer itself, and co-existing fatigue [5]. Depression and anxiety are also strongly correlated with cognitive problems [7]. In most patients, complaints of CRCI are mild to moderate [8], yet they can profoundly impact the quality of life [9]. Although some interventions are promising, no strategy is currently widely implemented or accepted to prevent CRCI in breast cancer patients [10].

Physical exercise during chemotherapy has been proposed as a strategy to prevent CRCI. Rodent studies describe various pathways via which exercise can benefit cognition, such as stimulating hippocampal neurogenesis [11, 12]. In non-cancer populations, most, but not all [13] studies report an association between higher levels of physical activity [14, 15] or exercise interventions [16, 17] and better cognitive outcomes. In cancer patients, most trials studied the effect of an exercise intervention after treatment (*i.e.,* in survivors), with most of them reporting positive effects on perceived cognition and not on tested cognition [18–21]. One small, randomized study (N = 25 per study arm) suggests that an unsupervised, home-based walking intervention during chemotherapy might mitigate self-reported CRCI directly after treatment [22]. Evidence from larger, well-conducted trials with longer follow-up times is lacking.

In this study, we evaluated the effect of an aerobic and resistance exercise intervention during adjuvant chemotherapy for breast cancer on cognitive testing and self-reported cognitive complaints measured, on average, 8.5 years after treatment. We hypothesized that exercise during chemotherapy, relative to usual care control, results in less CRCI years after treatment.

## Methods

### Setting and participants

The current analysis is part of the Pact-Paces-Heart study, a follow-up investigation of two previously performed randomized controlled trials (RCTs): the Physical Activity during Cancer Treatment (PACT) study and the Physical exercise during Adjuvant Chemotherapy Effectiveness Study (PACES). The design and results of the Pact-Paces-Heart study on cardiovascular outcomes (submitted), as well as results of the original studies, have been published elsewhere [23–25]. In brief, the PACT and PACES studies were conducted between 2009–2013 and included 204 and 230 non-metastasized breast cancer patients, respectively. In the PACT study, participants were randomized to either a supervised, moderate-to high-intensity exercise intervention or a control group. The intervention started six weeks after diagnosis with a fixed duration of 18 weeks. PACES’ design was comparable, except that there was a second intervention arm (a home-based, low-intensity exercise program), and both interventions of PACES started with the first cycle of chemotherapy and continued until three weeks post-treatment. Both studies collected data (e.g., physical fitness, muscle strength, and patient-related outcomes, including quality of life) at baseline, at the end of chemotherapy, and approximately six months after baseline. In PACT, physical activity levels were recorded by the Short Questionnaire to assess Health-enhancing physical activity (SQUASH) [26]. PACES used the Physical Activity Scale for Elderly [27]. In the follow-up study, physical activity was assessed via the SQUASH. Information on the exercise intervention, including the exercise dose and adherence, is provided in Appendix.

**Fig. 1:**
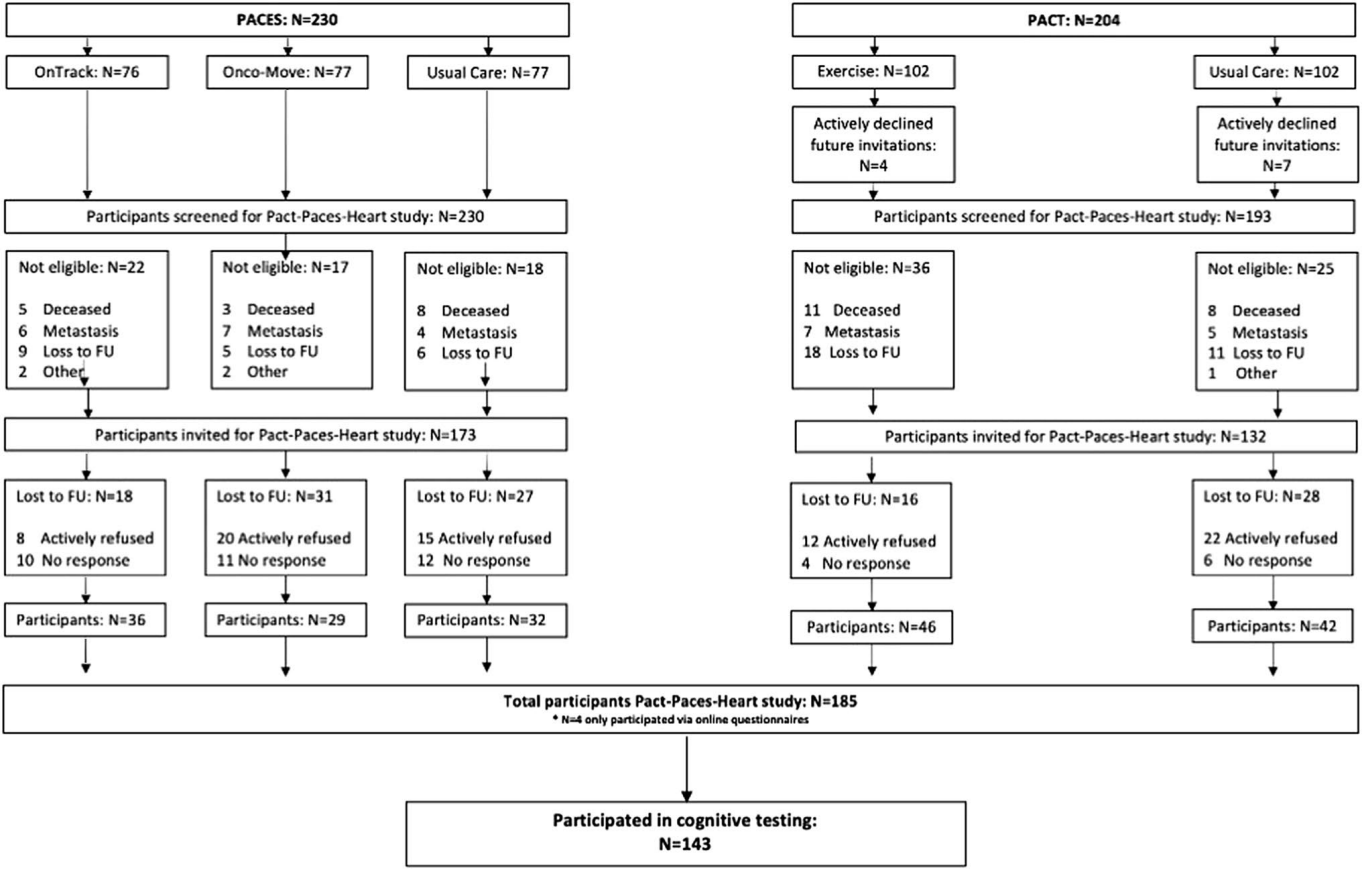
Flowchart of participants in the original PACT and PACES studies, and in the Pact-Paces-Heart study with cognitive testing

The parent study included 185 breast cancer survivors free of recurrent or metastasized cancer. Participants underwent physical measurements (*i.e.,* cardiac MRI, cardiopulmonary exercise test) and completed questionnaires. Participation in additional cognitive testing was optional. A detailed description of the flow of participants through the studies is provided in Fig. 1. The study was approved by the UMC Utrecht institutional review board and was registered with the International Clinical Trial Registry Platform (identifier NTR7247). All patients provided written informed consent.

### Cognitive outcomes

An overview of the data collection of cognitive outcomes at the different time points is provided in Table 1. Objective cognitive testing was performed using the online Amsterdam Cognition Scan (ACS). The ACS is a recently developed, self-administrated neuropsychological test battery that includes 11 computerized tests, based on traditional neuropsychological tests, in the following five cognitive domains: (1) learning and memory; (2) attention and working memory; (3) processing speed; (4) executive functioning; and (5) motor functioning [28]. Reliability and validity of the ACS have been previously described [28], and other oncology studies have used this tool to assess cognitive performance [20, 29].

**Table 1:** Overview of the data collection of cognitive outcomes at the different time points

Timepoint	Cognitive outcome		
	Subjective cognitive outcomes		Objective cognitive outcomes
	*EORTC QLQ-C30*	*MDASI*	*ACS*
Before treatment (T0)	X		
End of chemotherapy (T1)	X		
6-months after baseline (T2)	X		
8.5-years after baseline (T3)	X	X	X

*ACS* Amsterdam Cognition scan, *EORTC QLQ-C30* European Organization for Research and Treatment for Cancer Quality of Life Questionnaire, *MDASI* MD Anderson Inventory

Subjective cognitive complaints were assessed with the M.D. Anderson Symptom Inventory (MDASI) questionnaire [30], with additional questions from the MDASI multiple myeloma module [31. The cognitive questions of this module are not specific to multiple myeloma patients and have been previously related to tested cognition [32]. We included two questions on the severity of memory and attention problems and four questions on interference with daily life. Response options were on a 0–10 numeric scale, ranging from “not present” to “as bad as you can imagine” and “did not interfere” to “interfered completely” for the questions on severity and interference, respectively. From these raw scores, a mean subscale score for severity and interference was derived. A previous study reported good-to-excellent reliability, with Cronbach α coefficient values of 0.88 for the severity subscale and 0.91 for the interference subscale [31]. Also, in both the original studies and the follow-up study, all participants of the Pact-Paces-Heart study completed questionnaires on patient-reported outcomes, including the European Organisation for Research and Treatment of Cancer Quality of Life Questionnaire Core 30 (EORTC QLQ-C30) [33]. “This questionnaire included two questions on cognitive function”. Following the EORTC QLQ-C30 scorings manual, we calculated the score for the cognitive functional scale by averaging the item scores and linearly transforming this value to obtain a range from 0 to 100, where a higher score corresponds with better functioning.

### Statistical analysis

Numerical data are presented as mean ± standard deviation, and ordinal data or numerical data violating normality assumptions as median [min–max]. Using pre-defined criteria, all ACS entries indicative of poor test understanding, periods of participant distraction, or computer/network problems were identified and excluded. For those tests where higher scores corresponded with worse cognitive performance, we calculated the absolute median deviation per age category (≤ 40, 41–59, ≥ 59 years), and all entries > 3.5 units were considered outliers and removed from the database [34]. To contextualize the overall cognitive performance of our cohort, ACS scores in our sample were compared to normative data (based on 248 healthy adult controls) [35], and expressed as age-adjusted Z-scores per study arm. For the cognitive functioning scale of EORTC QLQ-C30, we interpreted our results compared to previously described normative data [36] and used a score of < 75 as the threshold for clinically relevant cognitive impairment [37].

We used intention-to-treat regression models with treatment allocation (moderate-to high-intensity exercise versus control) and cognitive outcomes as independent and dependent variables, respectively. All outcomes were modeled linearly, except for those expressed on an ordinal scale (i.e., the correct number of words or sequences), which were modeled via a modified Poisson regression (with a log-link) and expressed as relative differences with robust standard errors (sandwich estimates). For the linear models with non-normally distributed residuals (e.g., the MDASI questionnaire data where most participants reported near-zero scores), estimates and bias-corrected confidence intervals (CIs) were calculated using a bootstrapped distribution based on 10,000 replications [38, 39].

All models were adjusted for age, education (low versus middle or high), study (PACT versus PACES), currently receiving endocrine treatment, and cumulative doxorubicin equivalent dosage (with ratio doxorubicin: epirubicin = 1: 0.7 [40]). We additionally adjusted these models for baseline EORTC QLQ C30 scores. Analyses were repeated with changes in self-reported physical activity during chemotherapy, independent of treatment allocation, as the main independent variable. Change in physical activity was defined as the level of physical activity after the intervention (T1) minus the level of physical activity at baseline (T0) and expressed as a z-score, given that the two original studies used different physical activity questionnaires. Restricted cubic splines were used to evaluate the potential nonlinearity of the latter models. We considered a p-value > 0.05 for the non-linear term as no indication of nonlinearity. Last, we repeated the analyses with physical activity levels at follow-up, expressed as minutes/week as the primary independent variable.

All exercise analyses were limited to the moderate-to high-intensity and control group because the low-intensity group of PACES was too small (N = 20). In a sensitivity analysis, we added data from these low intensity participants to the moderate-to-high intensity exercise group. The data from the low-intensity group was also included in the analyses with self-reported physical activity, given that those analyses were irrespective of randomization. All analyses were performed with R studio software (version 4.3.0, Rstudio Inc., Boston, MA).

## Results

Of the 185 Pact-Paces-Heart study participants, 143 (N = 143/185; 77.3%) participated in the optional cognitive testing. The demographic characteristics of those who completed the cognitive testing were comparable to those who did not participate in the cognitive testing and the original study sample (Supplementary Table 1).

### Descriptive results

Of the 143 participants, 66 had been allocated to the moderate-to high-intensity exercise program and 57 to the control arm. Characteristics of these participants is presented in Table 2. Information on the 20 participants of the low-intensity arm is provided in Supplementary Table 2.

**Table 2:** Characteristics of Pact-Paces-Heart participants who completed the cognitive testing (N = 143)

	Control	Exercise
	N = 57	N = 66
Age, years	58.3 ± 7.6	58.9 ± 6.4
Original study		
PACT, %	31 (54.3)	39 (59.0)
PACES, %	26 (45.6)	27 (40.9)
Follow-up time, years	8.6 ± 1.2	8.4 ± 1.2
Education, %		
Low	6 (10.7)	2 (3.0)
Middle	22 (39.3)	27 (40.9)
High	28 (50.0)	37 (56.1)
Menopausal status, %		
Premenopausal	4 (7.0)	5 (7.6)
Postmenopausal	52 (91.2)	61 (92.4)
Unknown	1 (1.8)	0 (0.0)
Receptor status		
Triple negative	8 (14.0)	12 (18.2)
ER/PR + , HER2 +	8 (14.0)	11 (16.7)
ER/PR-, HER +	2 (3.5)	5 (7.6)
ER/PR + , HER-	39 (68.4)	38 (57.6)
Radiotherapy, %		
No RT	13 (22.8)	17 (26.2)
Left-sided	21 (36.8)	26 (40.0)
Right-sided	21 (36.8)	22 (33.8)
Unknown	2 (3.5)	0 (0.0)
Anthracyclines, %		
No anthracyclines	0 (0.0)	1 (1.5)
Doxorubicin	34 (60.7)	30 (46.2)
Epirubicin	22 (39.3)	34 (52.3)
Unknown	1 (1.8)	1 (1.5)
Cumulative dose AC, mg/m^2^*	241 (91–420)	235 (0–420)
Medication use, %		
Cardiovascular	9 (16.1)	15 (23.1)
Anti-diabetic	0 (0.0)	1 (1.5)
Statins	3 (5.4)	3 (4.6)
Endocrine treatment	11 (19.6)	7 (10.8)
Other	22 (39.3)	17 (26.2)
Any comorbidity, %	21 (37.5)	22 (33.8)

Presented as mean ± SD, median [min–max] or number (percentages)

^*^Calculated using Doxorubicin: Epirubicin ratio = 1: 0

*AC* anthracycline (equivalent0), *ER* estrogen, *HER* human epidermal growth factor receptor, *int*. intensity, *PR* progesterone, *RT* radiotherapy

The moderate-to high-intensity and control group were comparable in most characteristics (Table 2). The average age at the time of cognitive testing was 58 years, and the vast majority (> 90%) of the participants were post-menopausal. Half of the participants in the control arm and 56% of the women in the moderate-to high-intensity exercise group were highly educated. All participants, except one, received treatment with anthracyclines, with median doxorubicin (equivalent) dosages of 241 [91–420] mg/m^2^ and 235 [0–420] mg/m^2^, in the control and moderate-to high-intensity exercise group, respectively. At the time of cognitive testing, 11 (N = 11/57; 19.6%) control participants and 7 participants of the moderate-to high-intensity exercise program (N = 7/66; 10.8%) received endocrine therapy. Comorbidities were reported in 21 (N = 21/57; 37.5%) and 22 (N = 22/77; 33.8%), respectively.

Self-reported QLQ-C30 cognitive functioning scores are presented in Table 3. The median score before treatment was 83.3 [0–100.0] in the control and 83.3 [16.7–100.0] in the moderate-to high-intensity group, with self-reported impaired cognitive functioning in 24.6% (N = 14/57) and 39.4% (N = 26/66), respectively. After chemotherapy treatment, these percentages increased to 50.0%(N = 24/48) in the control arm and 53.0% (N = 35/66) in the moderate-to high-intensity exercise arm directly after the intervention. At the six-month follow-up, 40.4% (N = 21/52) of the control participants and 41.3% (N = 26/62) of the moderate-to high-intensity exercise group participants reported impaired cognitive function. At 8.5 years post-treatment, median scores were comparable between study arms; 83.3 [0–100.0] and 83.3 [16.7–100.0], respectively. However, at follow-up, more patients in the exercise group reached the threshold for cognitive impairment (N = 31/66; 47.0%) compared to control participants (N = 20/56; 35.7%).

**Table 3:** Cognitive functioning based on the EORTC QLQ C-30 in the original studies, and at follow-up

	Control (N = 57)		Exercise (N = 66)	
	Data missing (N, %)	Cognitive functioning	Data missing (N, %)	Cognitive functioning
Cognitive functioning				
Before treatment	0 (0)	83.3 [0–100.0]	0 (0)	83.3 [16.7–100.0]
End of chemotherapy	9 (15.8)	75.0 [16.7–100.0]	0 (0)	66.7 [16.7–100.0]
6-months after baseline	5 (8.8)	83.3 [16.7–100.0]	4 (4.5)	83.3 [0–100.0]
8.5-years after baseline	1 (1.8)	83.3 [0–100.0]	0 (0)	83.3 [16.7–100.0]
Cognitive functioning < 75, (%)				
Before treatment	0 (0)	14 (24.6)	0 (0)	26 (39.4)
End of chemotherapy	9 (15.8)	24 (50.0)	0 (0)	35 (53.0)
6-months after baseline	5 (8.8)	21 (40.4)	4 (4.5)	26 (41.3)
8.5-years after baseline	1 (1.8)	20 (35.7)	0 (0)	31 (47.0)

Presented as median [min–max] or number (percentages)

Scores on the ACS and the MDASI 8.5 years post-treatment are presented in Table 4. Based on the age-adjusted z-scores, participants in our study tended to score lower than healthy controls on the tests assessing learning and memory, attention and working memory, and motor functioning. Above average, although with wide/ non-significant confidence intervals, z-scores were observed for tests of the domain’s processing speed and executive functioning.

**Table 4:** Cognitive outcomes and corresponding age-corrected Z-scores per study arm

	Control (N = 57)		Exercise (N = 66)	
	Raw score	Age-adjusted Z-score* Beta (95% CI)	Raw score	Age-adjusted Z-score* Beta (95% CI)
Objective cognitive functioning (ACS)				
Learning and memory				
Wordlist Learning (words, N)	47.0 [27–74]	−0.37 (−0.63, −0.10)	47.0 [13–68]	−0.41 (−0.70, −0.12)
Wordlist Delayed Recall (words, N)	11.0 [6–15]	−0.17 (−0.43, 0.10)	10.0 [4–15]	−0.25 (−0.52, 0.02)
Wordlist Recognition (words, N)	29.0 [26–30]	NA^**^	29.0 [23–30]	NA^**^
Attention and working memory				
Box Tapping (correct sequences, N)	9.0 [5–12]	0.17 (−0.02, 0.17)	9.0 [6–12]	0.18 (0.03, 0.33)
Digit Sequences I (correct sequences, N)	10.0 [4–15]	−0.39 (−0.68, −0.10)	10.0 [6–16]	−0.13 (−0.38, 0.13)
Digit Sequences II (correct sequences, N)	8.0 [3–13]	0.11 (−0.35, 0.12)	8.0 [3–14]	−0.18 (−0.05, 0.42)
Processing speed				
Reaction Time (completion time, 10-ms)	31.6 ± 4.1	0.10 (−0.14, 0.35)	34.0 ± 5.3	0.25 (−0.53, −0.02)
Connecting the Dots I (completion time, ms)	37.5 ± 7.8	0.35 (−0.18, 0.51)	35.6 ± 6.7	0.57 (−0.74, 0.40)
Executive functioning				
Connecting the Dots II (completion time, ms)	62.0 ± 15.5	0.34 (0.10, 0.57)	61.1 ± 16.2	0.42 (−0.18, 0.67
Place the Beads (required moves, N)	27.5 [7–64]	0.17 (−0.08, 0.42)	23.5 [4–74]	0.39 (−0.16, 0.62)
Motor functioning				
Fill the Grid (completion time, ms)	68.3 ± 13.4	−0.02 (−0.32, 0.27)	70.7 ± 15.3	−0.20 (−0.51, 0.19)
Self-reported cognitive function (MDASI)				
Severity subscale (mean score)	1.50 [0–10]	NA	2.0 [0–9]	NA
Severity subscale classification (%)				
No symptoms	14 (25.0)	NA	16 (24.2)	NA
Mild symptoms	31 (55.4)	NA	34 (51.5)	NA
Moderate symptoms	6 (10.7)	NA	9 (13.6)	NA
Severe symptoms	5 (8.9)	NA	7 (10.6)	NA
Interference subscale (mean score)	0.33 [0–7]	NA	0.92 [0.00–7.3]	NA

Presented as mean ± SD or median [min–max]

^*^Compared to normative data of 248 non-cancer controls [35]

^**^Normative data was not available for this test, since this test has been updated since the original version

*ACS* Amsterdam Cognition Scan, *CI* confidence interval, *MDASI* MD Anderson Index, *ms* millisecond, *N* number, *NA* not applicable

For self-reported cognitive functioning (MDASI) 8.5 years after treatment, most patients reported none or mild symptoms with median scores of 1.50 [0–10] in control participants and 2.0 [0–9] in participants of the moderate-to high-intensity exercise program. Moderate or severe symptoms were reported by 6 (10.7%) and 5 (8.9%), 9 (13.6%), and 7 (10.6%) participants, respectively. Scores on the interference subscale were 0.33 [0–7] in the control group and 0.92 [0–7.3] in the moderate-to high-intensity exercise group.

### Effect of moderate-to high-intensity exercise on long-term tested and perceived cognition

We did not find any significant effect of moderate-to high-intensity exercise during chemotherapy on objective cognitive testing 8.5 years after treatment (Table 5). The result for the test Reaction Time significantly favored the control group (β per 10-ms = 1.87, 95%CI: 0.06; 3.69). Estimates for self-reported cognitive functioning (MDASI) tended to favor control participants, although the results were not statistically significant. The models with additional correction for baseline EORTC QLQ-C30 cognitive functioning scores yielded comparable results, except for the results for the Reaction Time test (β per 10-ms = 1.83, 95%CI: -0.01; 3.67). In the sensitivity analysis, where data of participants of the low-intensity exercise program were added to the intervention group, the result on the test Reaction was not significant anymore; β per 10-ms = 1.70, 95%CI: -0.03; 3.43, while the test on Digit Sequence II now significantly favored the exercise arm (relative difference per number of sequences of 1.14, 95%CI: 1.02, 1.26). The other models generated similar conclusions to those presented in Table 4 (data not shown).

**Table 5:** Effect of an exercise intervention during chemotherapy on tested and perceived cognitive functioning 8.5 years post-treatment

	Regression model	Estimate	Unadjusted (95%CI)*	Fully adjusted 95%CI)**
Objective cognitive testing (ACS)				
Learning and memory				
Wordlist Learning (words, N)	Poisson	Relative difference	0.99 (0.91, 1.07)	0.98 [0.91, 1.06]
Wordlist Delayed Recall (words, N)	Poisson	Relative difference	0.98 [0.89, 1.07]	0.97 [0.90, 1.06]
Wordlist Recognition (words, N)	Poisson***	Relative difference	1.00 [0.98, 1.01]	1.00 [0.99, 1.0
Attention and working memory				
Box Tapping (correct sequences, N)	Poisson	Relative difference	1.00 [0.93, 1.07]	0.99 [0.93, 1.04]
Digit Sequences I (correct sequences, N)	Poisson	Relative difference	1.06 [0.97, 1.16]	1.06 [0.96, 1.17]
Digit Sequences II (correct sequences, N)	Poisson	Relative difference	1.10 [0.98, 1.23]	1.11 [0.99, 1.24]
Processing speed				
Reaction Time (completion time, 10-ms)	Linear	Beta-coefficient	1.75 [0.01, 3.50]	**1.87 [0.06, 3.69]**
Connecting the Dots I (completion time, ms)	Linear	Beta-coefficient	−1.85 [−4.49, 0.79]	−2.00 [−4.36, 0.36]
Executive functioning				
Connecting the Dots II (completion time, ms)	Linear	Beta-coefficient	−0.85 [−6.74, 5.05]	−0.90 [−6.46, 4.67]
Place the Beads (required moves, N)	Poisson	Relative difference	0.90 [0.75, 1.08]	0.91 [0.76, 1.10]
Motor functioning				
Fill the Grid (completion time, ms)	Linear	Beta-coefficient	2.48 [−2.78, 7.73]	2.86 [−2.35, 8.06]
Self-reported cognitive function (MDASI)				
Severity subscale	Linear***	Beta-coefficient	0.32 [−0.61, 1.24]	0.39 [−0.57, 1.34]
Interference subscale	Linear***	Beta-coefficient	0.34 [−0.33, 1.01]	0.55 [−0.14, 1.24]

^*^High-intensity, supervised exercise versus control (ref), Onco-Move left out

^**^Adjusted for age, education level, study (PACT vs PACES), AC dose and current endocrine treatment (yes/no)

^***^Using a Bootstrapping distribution

*ACS* Amsterdam Cognition Scan, *CI* confidence interval, *MDASI* MD Anderson Index, *ms* millisecond, *N* number

### Association between physical activity levels and long-term cognition

An increase in physical activity level during chemotherapy was not associated significantly with objectively assessed or self-reported cognitive functioning years after treatment (Table 6). Similarly, physical activity levels at follow-up were not associated significantly with cognitive outcomes (relative differences range from 0.99 to 1.01, and beta coefficients per 0 to 0.07 per 10-min difference in reported physical activity, data not shown).

**Table 6:** Association between change in self-reported physical activity* during chemotherapy and tested and perceived cognitive functioning 8.5 years post-treatment

	Regression model	Estimate	Unadjusted (95%CI)	Fully adjusted 95%CI)^**^
Objective cognitive testing (ACS)				
Learning and memory				
Wordlist Learning (words, N)	Poisson	Relative difference	0.99 [0.94, 1.04]	0.99 [0.95, 1.02]
Wordlist Delayed Recall (words, N)	Poisson	Relative difference	0.99 [0.96, 1.03]	0.99 [0.95, 1.02]
Wordlist Recognition (words, N)	Poisson***	Relative difference	0.99 [0.98, 1.00]	0.99 [0.98, 1.00]
Attention and working memory				
Box Tapping (correct sequences, N)	Poisson	Relative difference	1.00 [0.97, 1.03]	1.00 [0.97, 1.03]
Digit Sequences I (correct sequences, N)	Poisson	Relative difference	1.01 [0.96, 1.06]	1.00 [0.96, 1.05]
Digit Sequences II (correct sequences, N)	Poisson	Relative difference	1.01 [0.96, 1.07]	1.01 [0.96, 1.06]
Processing speed				
Reaction Time (completion time, 10-ms)	Linear	Beta-coefficient	−0.31 [−1.23, 0.61]	−0.47 [−1.40, 0.46]
Connecting the Dots I (completion time, ms)	Linear	Beta-coefficient	1.02 [−0.42, 2.46]	1.14 [−0.05, 2.33]
Executive functioning				
Connecting the Dots II (completion time, ms)	Linear	Beta-coefficient	−0.67 [−3.99, 2.65]	−0.33 [−3.29, 2.63]
Place the Beads (completion time, ms)	Poisson	Relative difference	1.02 [0.95, 1.10]	1.00 [0.93, 1.08]
Motor functioning				
Fill the Grid (completion time, ms)	Linear	Beta-coefficient	1.27 [−1.27, 3.80]	1.42 [−0.90, 3.73]
Subjective cognitive complaints (MDASI)				
Severity of symptoms	Linear***	Beta-coefficient	0.09 [−0.34, 0.53]	0.11 [−0.34, 0.55]
Interference with daily living	Linear***	Beta-coefficient	−0.07 [−0.40, 0.25]	−0.09 [−0.42, 0.24]

^*^Change is defined as the sum of physical activity at the end of chemotherapy minus the sum of physical activity at baseline

^**^Adjusted for age, education level, study (PACT vs PACES), AC dose and current endocrine treatment (yes/no)

^***^Using a Bootstrapping distribution

*ACS* Amsterdam Cognition Scan, *CI* confidence interval, *MDASI* MD Anderson Index, *ms* millisecond, *N* number

## Discussion

In this follow-up study, we investigated the effect of exercise and physical activity on tested cognitive functioning and self-reported cognitive complaints in patients with breast cancer who had participated in one of two randomized clinical trials of exercise programs during their primary chemotherapy treatment approximately 8.5 years earlier. Overall, cognitive performance in some domains (particularly learning and memory) was mildly impaired compared to normative data. Most, but not all, participants reported low levels of perceived cognitive symptoms, with less interference in daily life. We observed no significant effects of moderate-to high-intensity exercise or being more physically active during chemotherapy on tested or perceived cognitive functioning years later, compared to non-exercise controls. Moreover, regardless of randomization, we found no significant association between those who reported higher physical activity levels at follow-up and (better) cognitive outcomes.

Most previous studies that have investigated the effects of exercise performed after the treatment of breast cancer on CRCI reported positive effects on (self-reported) cognitive outcomes [18–20] However, minimal evidence is available on the efficacy of exercise during chemotherapy on CRCI. From a mechanistic point of view, multiple pathways support the hypothesis of exercise-mediated neuroprotection, including increased resting brain-derived neurotrophic factor (important for various cellular processes such as neurogenesis), local changes in vascularization, and less neuroinflammation [41–43]. This biological rationale is supported by the results of a recent, large observational study of breast cancer patients undergoing adjuvant chemotherapy (N = 580), that found that higher self-reported physical activity levels before and during chemotherapy were associated with better perceived and objectively measured cognitive function after chemotherapy completion [44]. The only currently available trail that reports on the effectiveness of exercise during chemotherapy generated inconclusive results. This study randomizes breast cancer patients to either an unsupervised, home-based walking intervention during chemotherapy (N = 25) or usual care (N = 25) and found significantly higher levels of perceived cognitive complaints in the latter group but not in the exercise group [22]. There was some evidence for between-group differences (p interaction for study group x time: 0.05). Nevertheless, given the limited sample size and that objective cognitive functioning appeared to be unaffected, limited conclusions for clinical practice can be made from these results, and thus more robust evidence is needed.

Our results indicate that exercise during chemotherapy was not associated with tested CRCI years after treatment. This finding does not necessarily mean that there were no exercise effects directly after treatment, given that cognitive performance may change over time. The original PACES study reported an effect of exercise on self-reported cognitive complaints, based on the EORTC QLQ C-30, with an effect size of 0.33 [24]. A recent individual participant meta-analysis reported small effects of exercise post-treatment on self-reported cognitive functioning [45]. In a longitudinal, randomized study among breast cancer patients that studied the effects of self-affirmation(N = 160), perceived cognitive symptoms also varied over time. While the MDASI scores initially increased from baseline to the end of chemotherapy, at six months after chemotherapy, scores on the symptom subscale gradually decreased to an average of 2.10 ± 2.01 for patients in the control arm [46]. These findings have been corroborated by longitudinal neuroimaging studies documenting decreased cognitive performance during chemotherapy, with partial recovery [47] or even increased performance years after treatment in some patients [48]. Our study also observed above-average test scores in the domains of Processing Speed and Executive Functioning, but confidence intervals were wide. The current prevailing hypothesis is that the adult brain, although to a lesser extent than during childhood/adolescence, has the capacity to adapt to environmental changes and recover after disease by, for example, recruiting alternative neuronal circuits [49–51]. These neural plasticity processes are likely susceptible to cognitive training, such as memory training or speed tasks [52, 53]. If and to what extent participants compensated for cognitive impairments over time (with or without exercise) is an interesting topic for future research.

Based on the MDASI questionnaire, more than three-quarters of our study participants reported no or mild cognitive symptoms. Scores on the interference subscale were also low. Nevertheless, these results also indicate that a substantial proportion has moderate, or even severe, cognitive complaints years after treatment. The latter aligns with the findings on the EORTC QLQ-C30 questionnaire, in which 40% of the participants reached the threshold for clinically relevant cognitive impairment. The MDASI questionnaire assesses cognitive symptoms and their interference in the past 24 h, while the EORTC QLQ-C30 cognitive functioning is based on the past week. Subscales on the former instrument are also based on more questions with more extensive scoring ranges, which might have allowed for reporting more details on cognitive complaints. Prior research has reported a good (ρ = 0.69) and a moderate correlation (ρ = 0.49) between the MDASI symptom and interference subscale and the EORTC QLQ-C30 cognitive functioning scale, respectively [31].

Our current study was designed as a post-hoc, post-trial follow-up (FU) investigation of two original randomized trials (*i.e.,* the PACT and PACES study). This post-trial FU design allowed for pragmatically investigating the effect of exercise and physical activity on long-term CRCI in a relatively large sample of breast cancer survivors. Post-trial FU studies can effectively detect persistent or even enhanced treatment effects years after completion of the original trials, sometimes referred to as the ‘legacy effect’ [54, 55]. Also, delayed adverse effects, which take years or even decades to become clinically apparent, can be detected by post-trial FU [56], as exemplified by former studies documenting the cardiotoxic properties of high-dose thoracic radiotherapy [57–59]. However, by design, post-trial FU studies may be susceptible to selective response/drop-out, especially with more extended periods of FU. In the context of our research, it is conceivable that breast cancer survivors who were originally randomized to the intervention program, or controls who are currently relatively fit and free of symptoms, were more willing to participate in our follow-up trial (and especially in additional, optional cognitive tests). Indeed, we included slightly more participants who were originally randomized to the exercise arm than to the control arm; 46% (n = 66/143) versus 40% (n = 57/143), respectively. The proportion of control participants in this follow-up study with cognitive impairment before treatment was lower (n = 14/57; 24.6%) than the proportion in the exercise group (n = 26/66; 39.4%). Thus, a selective response may have diluted our results to a certain extent, although we observed no significant difference in demographic characteristics between those who participated in our FU study and those who did not. A recently published systematic review recommended using registries and data linkage as the most effective approach for post-trial FU studies [56]. Given that such an approach is not possible for endpoints such as patient-reported outcome measures, we suggest, as we did in the first follow-up of PACT [25], that future randomized studies embed a question in the informed consent that allows for potential future data linkage and study invitation to facilitate future post-trial FU investigations.

Our study has several strengths and limitations in addition to those related to the post-trial FU design. A strength is the combination of objectively tested and self-reported cognitive outcomes in our study, given that these outcomes often are not highly correlated and might measure different constructs of CRCI [60, 61]. An additional limitation is that, apart from the EORTC QLQ-C30 questionnaire, cognitive outcomes were not included in the original trials, and thus we cannot correct for baseline values of the outcome. Also, not all participants experienced cognitive impairment prior to the intervention, with half of them reaching the threshold for clinically impaired cognitive functioning (Table 3). This means that the other half was unlikely to benefit from the intervention, which presumably limited our ability to study the effectiveness of the exercise program on cognitive functioning. Last, our exercise program was not tailored specifically to address cognitive complaints and was perhaps not the most optimal program for that purpose. A previous meta-analysis indicated that, in addition to aerobic and resistance exercise, non-western traditional modes of exercise, such as Tai Chi or yoga, are at least equally effective in improving cognitive functioning [62]. Thus, a multicomponent exercise program incorporating more holistic exercises might confer greater improvement in cognition functioning.

In conclusion, in this pragmatic follow-up study, we observed that exercising or being more physically active during chemotherapy was not associated with tested and perceived cognitive functioning years after treatment for breast cancer. Similarly, higher levels of reported physical activity at follow-up were not associated with better cognitive outcomes. Future prospective studies are warranted to investigate the complex relationship between exercise and enhanced physical activity among breast cancer patients who have experienced treatment-related impairment in their cognitive functioning.

### Electronic supplementary material

Below is the link to the electronic supplementary material.

Supplementary file1 (DOCX 16 KB)

## Data Availability

The data supporting this study’s findings are available from the corresponding author, AM, upon reasonable request.

## Appendix: Exercise intervention of PACT and PACES

The moderate-to high-intensity exercise programs of the PACT and PACES studies comprised two sessions of aerobic and resistance exercise per week under the supervision of a trained physical therapist. The programs were tailored to the participants’ exercise capacity. The attendance rate was 81% and 73% for PACT and PACES, respectively [23, 24]. In addition, participants were encouraged to be physically active for at least 30 min per day on the remaining days of the week. Participants allocated to the low-intensity, home-based exercise program of PACES were coached by a trained oncology nurse and received written information on physical activity. PACT and PACES control participants were asked to maintain their pre-treatment physical activity levels.
