# Supplementary material for: Effects of physical exercise during adjuvant chemotherapy for breast cancer on long-term tested and perceived cognition: results of a pragmatic follow-up study
Source: Breast Cancer Res Treat. 2024 Jan 29;205(1):75–86. doi: 10.1007/s10549-023-07220-7 (PMC11062992; doi:10.1007/s10549-023-07220-7)
Supplement: Supplementary file 1 — Supplementary file1 (DOCX 16 KB) [file 10549_2023_7220_MOESM1_ESM.docx]

**Supplementary table 1. Demographic characteristics of the original PACT (N=204) and PACES (N=230) participants, and participants in the follow-up study.**

Presented as mean ± SD, median [min-max] or number (percentages)

**Original PACT and PACES Pact-Paces-Heart study (N=185)**

cognitive testing no cognitive testing N=434 N=143 N=42

**-----------------------------------------------------------------------------------------------------------------------------------------------------------------**Age, years 50.1 ± 8.6 50.3 ± 7.2 50.8 ± 9.3

Original study, %

PACT 204 (47.0) 70 (49.0) 18 (42.9)

PACES 230 (53.0) 73 (51.0) 24 (57.1)

Randomization, %

Exercise: high-int 178 (41.0) 66 (46.2) 16 (48.5)

Exercise: low-int 77 (17.7) 20 (14.0) 9 (21.4)

Control 179 (41.2) 57 (40.0) 17 (40.4)

Education, %

Low 40 (9.4) 9 (6.3) 0 (0.0)

Middle 176 (41.4) 58 (40.8) 12 (28.6)

High 209 (49.2) 75 (52.8) 30 (71.4)

Receptor status, %

Triple negative 78 (18.0) 23 (16.1) 6 (14.3)

ER/PR+, HER2+ 73 (16.8) 23 (16.1) 8 (19.0)

ER/PR-, HER+ 23 (5.3) 8 (5.6) 4 (9.5)

ER/PR+, HER- 260 (59.9) 89 (62.7) 24 (57.1)

Cumulative dose AC, mg/m^2^* 240 [0-431] 240 [0-431] 240 [176-420]

Pre-treatment cognitive functioning

EORTC QLQ-C30 83 [0-100] 83 [0-100] 83 [0-100]

EORTC QLQ-C30<75, % 142 (32.8) 50 (35.0) 13 (31.0)

**------------------------------------------------------------------------------------------------------------------------------------------------------------------**

Abbreviations: AC = anthracycline (equivalent0, ER = estrogen, EORTC QLQ C-30: European Organization of Research and Treatment of Cancer Quality of Life, HER = human epidermal growth factor receptor, PR = progesterone, RT = radiotherapy.

**Supplementary Table 2. Characteristics of low-intensity exercise group of PACES (N=20)**

Presented as mean ± SD, median [min-max] or number (percentages)

**Low-intensity exercise**

**N=20**

**---------------------------------------------------------------------------------------------------------**

Age, years 59.8 ± 9.3

Original study

PACT, % 0 (0.0)

PACES, % 20 (100.0)

Follow-up time, years 9.2 ± 0.8

Education, %

Low 1 (5.0)

Middle 9 (45.0)

High 10 (50.0)

Menopausal status, %

Premenopausal 2 (10.0)

Postmenopausal 18 (90.0)

Unknown 0 (0.0)

Receptor status

Triple negative 3 (15.0)

ER/PR+, HER2+ 4 (20.0)

ER/PR-, HER+ 1 (5.0)

ER/PR+, HER- 12 (60.0)

Radiotherapy, %

No RT 8 (40.0)

Left-sided 5 (25.0)

Right-sided 5 (25.0)

Unknown 2 (10.0)

Anthracyclines, %

No anthracyclines 0 (0.0)

Doxorubicin 15 (78.9)

Epirubicin 4 (21.1)

Unknown 1 (5.0)

Cumulative dose AC, mg/m^2^* 292 (196-431)

Medication use, %

Cardiovascular 4 (20.0)

Anti-diabetic 1 (5.0)

Statins 2 (10.0)

Endocrine treatment 1 (5.0)

Other 1 (5.0)

Any comorbidity, % 5 (25.0)

**---------------------------------------------------------------------------------------------------------**

* Calculated using Doxorubicin : Epirubicin ratio = 1 : 0.

Abbreviations:

AC = anthracycline (equivalent0, ER = estrogen, HER = human epidermal growth factor receptor, int. = intensity, PR = progesterone, RT = radiotherapy.
